# Experienced Poor Lighting Contributes to the Seasonal Fluctuations in Weight and Appetite That Relate to the Metabolic Syndrome

**DOI:** 10.1155/2009/165013

**Published:** 2009-06-07

**Authors:** Sharon Grimaldi, Ani Englund, Timo Partonen, Jari Haukka, Sami Pirkola, Antti Reunanen, Arpo Aromaa, Jouko Lönnqvist

**Affiliations:** National Institute for Health and Welfare, P.O. Box 30, 00271 Helsinki, Finland

## Abstract

We tested which environmental, social, lifestyle, and health related factors of the individual contribute to the seasonal variations in mood and behavior and whether these influence the risks of the metabolic syndrome and major depressive disorder, both conditions having a high prevalence in industrialized populations. 5480 individuals, representative of the general population aged 30 and over in Finland, were assessed for metabolic syndrome using the ATP-III criteria, gave a self-report of seasonal variations in mood and behavior, and were interviewed for mood, anxiety, and alcohol use disorders using the DSM-IV criteria. The seasonal variations in mood and behavior have a metabolic factor composed of weight and appetite, and greater loadings on this factor increased the risk of metabolic syndrome (odds ratio of 1.18, 95% confidence interval of 1.10 to 1.26). Self-reports of lighting experienced as poor at home contributed to scores on the metabolic factor (*t* = 4.20, *P* < .0001). Lighting conditions and their dynamics may serve as a measure for intervention in order to influence the seasonal metabolic signals and in the end to prevent the metabolic syndrome.

## 1. Introduction

Health-related seasonal phenomena, such as infections and indices of cardiovascular or respiratory disease, are common [1]. They may have a substantial impact on health, longevity, and health-related quality of life instantly within a population at large or regularly during the individual's life year after year [2, 3]. Improved public health typically reduces such seasonal fluctuations.

Surveys indicate that the seasonal variations in mood and behavior tend to rise with increasing latitude, or following northward migration [4]. Individuals who spend less time outdoors tend to have more depressive symptoms [5]. This may come to a head at northerly regions where people tend to experience problems with sleep length, mood, and energy level during winter [6]. These problems may emerge, in addition to psychosocial distress, due to the shortage of sunlight or inadequate lighting and subsequent errors in the circadian clockwork regulating both daily and seasonal rhythms [7–11].

Seasonal variations in sleep length, appetite, and energy level in particular may activate negative thoughts in individuals with low self-esteem and poor social support so that the depressive episode is precipitated earlier in the autumn and is of longer duration [12]. Having experienced more numerous negative life events or low levels of social support and being nonnative or a woman are predictive of affective disorder with the seasonal pattern [13]. Although the seasonal patterns of depressive symptoms are fairly frequent, seasonal affective disorders appear to be less common than this would suggest [14, 15].

Co-occurrence of the metabolic syndrome is frequent as disturbances in metabolic networks, in glucose metabolism for instance, are implicated and often form a key component in major depressive disorder [16, 17]. Earlier, we found that the metabolic syndrome is associated with higher global scores on the seasonal variations in mood and behavior, and with the seasonal fluctuation in weight in particular [18].

### 1.1. Study Aims

Herein, we wanted to analyze which environmental, social, lifestyle, and health related factors of the individual contribute to the seasonal variations in mood and behavior at population level using a representative sample of all the Finns aged 30 and over for the analysis. Since the assessment of seasonal variations in mood and behavior gives us an estimate of the circadian clockwork and there are disturbances in the metabolic networks [17] as well as disruptions of the circadian rhythms [19] in mood disorders, we in addition wanted to see whether there are metabolic or mood components in the seasonal variations in mood and behavior and, if there were, whether they are associated with the risk of the metabolic syndrome and that of major depressive disorder.

## 2. Methods

This study was part of a nationwide health interview and examination survey, the Health 2000 Study, which was carried out in Finland, a north-eastern (60–70°N, 20–31°E) European country with about 5 million inhabitants, from September 2000 to June 2001. The study project was coordinated by the National Public Health Institute and implemented in collaboration with the Ministry of Social Affairs and Health. The two-stage stratified cluster sampling design was planned by Statistics Finland. The sampling frame comprised adults living in mainland Finland. This frame was regionally stratified according to the five university hospital regions, or catchment areas, each containing roughly one million inhabitants. From each university hospital region, 16 health care districts were sampled as clusters (80 health care districts in the whole country, including 160 municipalities, or groups of municipalities with joint primary care). The 15 biggest health care districts in the country were all selected in the sample, and their sample sizes were proportional to population size. The remaining 65 health care districts were selected by systematic probability proportional to size sampling in each stratum, and their sample sizes (ranging from 50 to 100) were equal within each university hospital region, the total number of persons drawn from a university hospital region being proportional to the corresponding population size. The 80 health care districts were the primary sampling units, and the ultimate sampling units were persons who were selected by systematic sampling from the health centre districts. From these 80 health care districts, a random sample of individuals was drawn using the data provided by Population Register Centre. Its population information system contains the official information for the whole country on the Finnish citizens and aliens residing permanently in Finland.

### 2.1. Sample

All the persons aged 30 and over (*n* = 8028) who were identified from the nationally representative sample by The Social Insurance Institution of Finland were contacted in person. Interviewers attended training sessions on the specific themes that were to be covered in the computer-assisted interviews. Of the final sample of 7979 persons, 6986 (88%) were interviewed at home or institution face to face and 6354 (80%) attended the health status examination in a local health center or equal setting, while 416 took part in the health status examination at home or in an institution. Overall, 84% participated either in the health status examination proper or in the examination at home. All the methods are reported more in detail on the Internet site of the Health 2000 (for details, see http://www.ktl.fi/health2000).

During the interviews, the background and health related information, and data on living habits were collected. As part of the interview, experienced lighting levels at home (poor or not poor) and lighting experienced as insufficient at work (not present or no problem, troubles to some extent, troubles quite a lot, or troubles exceedingly) were scored. Both the former score and the sum of the scores on the two items were categorized for the analysis to serve as a variable of the self-reported adequacy of lighting. The participants were handed out a questionnaire to have information on a range of symptoms, the use of time and leisure activities, physical exercise, alcohol consumption, mental health with the 12-item General Health Questionnaire (GHQ-12), and depressive symptoms with a modified Beck Depression Inventory (BDI) for example.

During the health status examination the waist circumference (in centimeters) was measured on the naked waist at the end of light expiration while the examinee was standing. The measurement was taken half way between the iliac crest and the lowest rib. The pelvic circumference (in centimeters) was measured at the broadest point of the pelvis. The height (in centimeters) and weight (in kilograms) were also measured, and the body-mass index (BMI) was calculated. To the end, a nurse checked that the first questionnaire had been filled in and the participants were handed out another questionnaire which was to be filled in at home and mailed thereafter back. This second questionnaire retrieved data for instance on the seasonal variations in mood and behavior with a modified Seasonal Pattern Assessment Questionnaire (SPAQ).

### 2.2. Assessment of Depressive, Anxiety, and Alcohol Use Disorders

The diagnostic mental health interview was performed at the end of the comprehensive health examination. The computerized version of the CIDI (M-CIDI) was used. The program uses algorithms to meet the DSM-IV criteria and allows the estimation of DSM-IV diagnoses for major disorders [20]. The translation of the M-CIDI into Finnish was made pairwise by psychiatric professionals and revised by others. The official Finnish translation of the DSM-IV classification was used as a basis for formulating the interview. The process included consensus meetings, third expert opinions, an authorized translator’s review, and testing with both informed test subjects and unselected real subjects [21]. Interviews were performed to determine the 12-month prevalence rates of major depressive episodes and disorder, dysthymia, general anxiety disorder, panic disorder with or without agoraphobia, social phobia, alcohol abuse and dependence, and other substance dependence and abuse. These were grouped into categories of depressive, anxiety and alcohol use disorders. Comorbid cases were defined as persons having suffered from any of these disorders from more than one category within the past 12 months.

### 2.3. Assessment of the Metabolic Syndrome

Metabolic syndrome was assessed using the US Adult Treatment Panel III of the National Cholesterol Education Program (NCEP-ATPIII) criteria [22] and defined as having at least three of the following components: the fasting blood glucose level was 6.1 mmol/L or higher, the blood pressure was high (systolic pressure was 130 mmHg or more or diastolic pressure was 85 mmHg or more), the serum triglycerides level was 1.7 mmol/L or higher, the serum high-density lipoprotein cholesterol level was lower than 1.0 mmol/L for men or lower than 1.3 mmol/L for women, or the waist circumference was 102.1 cm or more for men or 88.1 cm or more for women.

### 2.4. Self-Report Items

As part of the assessment, the participants filled in items concerning their leisure time exercise, alcohol use during the past 12 months, social activities, and activities spent outdoors. The intensity of exercise habits was categorized as follows: low (no strenuous exercise such as reading, watching, television or handicraft), medium (lightly strenuous exercise such as walking or bicycling for four or more times a week), keep-fit (fitness training for three or more hours a week), and sport (sports for several times a week). The frequency of alcohol use was categorized as follows: none, low (once to six times a year), medium (once to four times a month), and high (twice to seven times a week). The frequencies of social activities (meeting relatives, friends, or neighbors) and activities spent outdoors (exercise, hunting, fishing, gardening, or other outdoor recreation) were categorized as follows: low (less than once a year), medium (once a year to twice a month), and high (once to seven times a week).

The 12-item GHQ evaluates whether the individual complains about a recent symptom or behavior. It is a valid screening tool for and a measure of the psychological symptoms and mental health at population level in a range of settings and cultures, especially of feelings of anxiety and depression [23, 24]. It was scored on a Likert-like scale (less than usual, no more than usual, rather more than usual, or much more than usual), yielding a sum score ranging from 0 to 36. According to the analysis of data derived from the Health 2000 Study, the threshold value of 4 was taken to indicate ill health (the scores of 0 to 4 assigned as low and those of 5 to 36 as high).

The behavioral manifestation and symptom intensity of depression were assessed as self-report by using a modification of the 21-item BDI [25] as adapted and validated for the Finnish population (for further information, see http://www.kela.fi), with a sum score ranging from 0 to 55. The modified questionnaire was thereafter applied for using the cut-points of 9 and 18 (the scores of 0 to 9 assigned as low, those of 10 to 18 assigned as medium, and those of 19 to 55 as high).

The items of seasonal variation in mental health were taken and adapted from the SPAQ [26]. Two modifications were made to the original scoring as follows. Each of the six items of sleep length, social activity, mood, weight, appetite, and energy level was scored from 0 to 3 (none, slight, moderate, or marked change), not from 0 to 4 (none, slight, moderate, marked, or extremely marked change), with the sum or global seasonality score (GSS) ranging from 0 to 18. Next, the SPAQ poses a question: “If you experience changes with the seasons, do you feel that these are a problem for you?” This item was scored from 0 to 4 (none, mild, moderate, marked, or severe problem), not from 0 to 5 (none, mild, moderate, marked, severe, or disabling problem). The questionnaire was translated into Finnish and then back-translated in order to revise the linguistic accuracy.

### 2.5. Laboratory Tests

All participants had been asked to come to the health status examination fasting and without drinking on the same day. In the laboratory, a nurse recorded how these instructions had been followed and then took the blood samples. The samples were centrifuged at the examination site and placed into deep freezers at −20°C before they were transferred within one week to the National Public Health Institute and stored in deep freezers at −70°C.

Routine fasting laboratory tests included the concentrations of blood glucose and those of serum total cholesterol and triglycerides (Glucose Hexokinase, Cholesterol CHOD PAP and Triglycerides GPO PAP, Olympus System Reagent, Germany), those of high-density lipoprotein (HDL) cholesterol and low-density lipoprotein (LDL) cholesterol (HDL-C Plus and LDL-C Plus, Roche Diagnostics GmbH, Germany), and those of gamma-glutamyltransferase (GGT) and uric acid (IFCC/ECCLS and URIKAASI PAP, Konelab, Thermo Electron Oy, Finland).

### 2.6. Ethics

The National Public Health Institute coordinated and implemented the study project in collaboration with the Ministry of Social Affairs and Health. It provided a written informed consent to each participant, giving a full description of the protocol before signing it. The procedures were done according to the ethical standards of the responsible committee on human experimentation and with the Declaration of Helsinki, its amendments and revision.

### 2.7. Data Analysis

To start with, since we assessed the seasonal variations in mood and behavior with a modified questionnaire, we wanted to test its psychometric properties. Separate factor analyses were performed for the whole sample and four within-categories each (no diagnosis or depressive or anxiety or alcohol use disorder). The factor matrix was calculated using the maximum likelihood principle, and the standard orthogonal Varimax rotation was computed in order to examine the degree of correlation among factors using the RELIAB module of the Survo MM program, release 2 (http://www.survo.fi/mm/english.html).

Thereafter, our data were weighted to take into account the sampling design, and to reduce the bias due to nonresponse. The R project for Statistical Computing (R, version 2.2.1) was applied for, and its survey Package, available through the Comprehensive R Archive Network family of internet sites (http://www.r-project.org/), was run for the analysis of our data derived from a complex (a cluster-sampled, stratified, and unequally weighted) survey design using survey-weighted generalized linear models.

First, for the univariate regression models explaining the factor scores on the SPAQ, the following 30 explanatory variables were tested as a screen: the sex, longitude of residence, latitude of residence, area of living in two categories (the southern or northern part of Finland), region of residence in five categories (the five university hospital regions), age in four categories (30 to 45, 46 to 60, 61 to 75 or 76 to 99 years), education in three categories (low, middle, or high), marital status in two categories (living alone or with someone), physical exercise, alcohol use, social activities, activities spent outdoors, the experienced lighting levels at home, the self-reported adequacy of lighting at work, the GHQ-12 sum score, the BDI sum score, waist circumference, pelvic circumference, concentrations of the laboratory test parameters (glucose, glucose homeostasis assessment of insulin resistance index, glucose homeostasis assessment of islet beta-cell function index, total cholesterol, HDL cholesterol, LDL cholesterol, triglycerides, GGT, and uric acid), the metabolic syndrome, major depressive disorder, and the diagnosis of depressive or anxiety or alcohol use disorder or their combination. Only those explanatory variables which had statistical significance in the univariate regression models were included in the subsequent multivariate regression models.

Next, to elucidate whether the factor scores contribute to the risks, the assessment of the metabolic syndrome and major depressive disorder was taken as the dependent variable for the two multivariate logistic regression models.

To finalize with, the GSS and the extent of the seasonal variations experienced as a problem were taken as the dependent variable for the two multivariate regression models. The former was log transformed, the latter was binomial (a problem to any extent or not a problem).

In the end, to control the number of tests calculated, we applied Bonferroni correction for the level of significance.

## 3. Results

Of the 8028 participants (4391 women, 3637 men), 6696 were assessed for the metabolic syndrome, 6161 gave a self-report of whether the seasonal variations in their mood and behavior were a problem, and 5480 were interviewed with the M-CIDI for diagnosis.

### 3.1. Factor Analysis of the Seasonal Variations in Mood and Behavior

Our analysis of the scores on the GSS being composed of the six items found that a two-factor solution made the best match with the covariation, so our analysis disagreed with the assumption of a single global factor. The factor one (weight, appetite) explained 53%; whereas the factor two (sleep length, social activity, mood, energy level) explained 89% of the variance in the GSS. The former was assigned as the metabolic factor; whereas the latter was assigned as the mental factor. The calculation of reliabilities yielded Cronbach’s alpha of 0.81 and the all-item unweighted sum of 0.85 according to a general model with the assumption that measurement errors may correlate. A similar factor pattern of the SPAQ was found for each diagnostic category, with the highest loadings on the two factors being produced by the seasonal variations in appetite and mood for those with no diagnosis (*n* = 3857), weight, and energy level for those with depressive disorder (*n* = 360), appetite, and energy level for those with alcohol use disorder (*n* = 226), and weight and mood for those with anxiety disorder (*n* = 219), respectively. The psychometric properties of the modified SPAQ were good and considered as a valid self-report rating scale for the seasonal variations in mood and behavior in a general population aged 30 and over. The loading values on the two factors were then computed for each individual.

### 3.2. Explanatory Variables for the Metabolic and Mental Components

The loading values for the two factors identified of the SPAQ were taken as the dependent variables for the univariate regression models of the metabolic and mental components of the seasonal variations in mood and behavior. Of the 30 explanatory variables tested, the sex, the self-reported adequacy of lighting at home, the GHQ-12 sum score, the BDI sum score, waist circumference, pelvic circumference, the metabolic syndrome, and major depressive disorder contributed significantly to loading on the metabolic factor, whereas the sex, the GHQ-12 sum score, the BDI sum score, major depressive disorder, and the diagnosis of depressive or anxiety or alcohol use disorder or their combination contributed significantly to loading on the mental factor (Table 1).

In the multivariate regression models including the explanatory factors of significance in the univariate models, the sex, the self-reported adequacy of lighting at home, and waist circumference remained as significant predictors for loading on the metabolic factor, whereas the BDI sum score, the GHQ-12 sum score, and the sex remained to be of significance to loading on the mental factor.

The significant contribution of the metabolic syndrome and major depressive disorder to the metabolic factor gave us a rationale to analyze the risk of the two conditions more in detail.

### 3.3. Risk Assessment of the Metabolic Syndrome

The risk of the metabolic syndrome (*n* = 2105) was analyzed. First, univariate models were computed after which on the basis of the univariate models we formulated the multivariate model including the eight significant determinants only (Table 2). In this multivariate model, the age and loading on the metabolic factor remained to be in a significant association with the metabolic syndrome.

### 3.4. Risk Assessment of Major Depressive Disorder

The risk of major depressive disorder (*n* = 275) was analyzed. First, univariate models were computed, and thereafter on the basis of the univariate models we formulated the multivariate model including the seven significant determinants only (Table 3). In this multivariate model, the associations of the GHQ-12 and BDI sum scores with major depressive disorder were still significant.

### 3.5. Explanatory Analysis of the Seasonal Variations in Mood and Behavior

To translate back the report of the seasonal variations in mood and behavior, we wanted to elucidate which factors have relevance to the degree of these changes. To that end, we first computed the univariate models. Of the 30 explanatory variables tested, the sex, education, the GHQ-12 sum score, the BDI sum score, pelvic circumference and major depressive disorder contributed to the GSS in a significant way. Next, we formulated the multivariate model including these six significant determinants only. Of them, in a ranking order, the BDI sum score, the sex, education, pelvic circumference, and the GHQ-12 sum score were still significant determinants of the intensity of the seasonal variations in mood and behavior (Table 4).

The significant contribution of the diagnosis of depressive or anxiety or alcohol use disorder or their combination to the mental factor score gave us a rationale to analyze the comorbid conditions in relation to the report of the seasonal variations in mood and behavior. Subjects having depressive or anxiety or alcohol use disorder or their combination had significantly greater seasonal variations than those having no diagnosis (Table 5). Our item-focused analysis of the seasonal variations in mood and behavior found that the seasonal variations in the sleep length, weight, and appetite were significantly greater in participants with depressive disorder only, as compared with those having no diagnosis. The seasonal fluctuation in social activity was the greatest in participants with anxiety disorder only. However, individuals having a comorbid condition had the highest score on each item, except on the seasonal fluctuation in mood. Moreover, it is of note that individuals having a comorbid condition had the greatest ratio of the metabolic to mental factor scores, followed by participants having anxiety or depressive disorder only, whereas the ratio was antithetical in those having alcohol use disorder only.

### 3.6. Explanatory Analysis of the Seasonal Variations Experienced as a Problem

Because the intensity of changes in mood and behavior may not match with the extent of a problem experienced due to the seasonal variations, we wanted to contrast these two in our analysis. First, we computed the univariate models. Of the 30 explanatory variables tested, the sex, marital status, the self-reported adequacy of lighting at home, the GHQ-12 sum score, the BDI sum score, major depressive disorder, and the diagnosis of depressive or anxiety or alcohol use disorder or their combination contributed significantly to the extent of a problem experienced due to seasonal variations in mood and behavior. Second, we formulated the multivariate model including these seven significant determinants only. Of them, in a ranking order, the BDI sum score and the GHQ-12 sum score were still significant determinants (Table 6). Moreover, of the comorbid conditions, depressive and alcohol use disorders in particular had a significant contribution.

## 4. Discussion

Our key finding herein is that the self-reported adequacy of lighting at home contributed to scores on the metabolic factor of the seasonal variations in mood and behavior. Further, we found that the loadings on the metabolic factor rather than the global seasonality scores link to the risk of the metabolic syndrome. In other words, the metabolic factor of the seasonal variations in mood and behavior is a key to the metabolic syndrome. Moreover, our results give support to the hypothesized links between the metabolic and circadian cycles and extend these links to include relationships between metabolism and mood.

The routine seasonal variations in weight and appetite which contribute most to the metabolic factor may be regarded as a seasonal metabolic signal. To visualize the significance, there are 1 611 005 persons having the seasonal fluctuation in weight and 1 379 542 persons having the seasonal fluctuation in appetite among the Finns aged 30 and over, or in other words, 49.5% and 42.4% of the population. Moreover, 56.1% and 46.6% of the individuals having the metabolic syndrome have seasonal variations in their weight and appetite, respectively. The female sex, lighting experienced as poor at home, and bigger waist circumference are in turn associated with the metabolic factor.

Of these three significant determinants, the relative shortage of light indoors may trigger the seasonal fluctuation in appetite and the subsequent change in weight during the fall when the length of the day or photoperiod starts decreasing. Poor lighting as a trigger makes sense, since lighting conditions during the morning hours in specific give stimuli to the master circadian clock to accelerate its period and thereby to correct its tendency towards delays. The self-reported adequacy of lighting at home may also make a difference as it does contribute not only to a major degree to the metabolic factor, but also to a minor extent to the experience of seasonal variations as a problem. Because artificial lighting conditions can be manipulated easily, they provide a way to intervention and health promotion. Intelligent housing solutions using computer-driven operations for the design of lighting levels and schedules during the day may be of benefit to individuals or groups having seasonal variations in mood and behavior or those who experience these changes as a problem.

The female sex, higher education, and bigger pelvic circumference contribute to the intensity of seasonal variations in mood and behavior that is associated with the increased risk of the metabolic syndrome. These significant associations indicate that both inherited genetic and individual environmental effects play a role. The report of whether the seasonal variations are to be experienced as a problem then depends on the current intensity of depressive symptoms and the actual feeling of mental well-being.

Since the seasonal variations or cycles are generated by the master circadian pacemaker or clock whose function produces the circadian rhythms as well [27], the assessment of seasonal variations in mood and behavior gives us an estimate of the circadian clockwork. The master circadian clock which is located in the suprachiasmatic nuclei of the anterior hypothalamus in the brain receives stimuli and feedback from the light-dark transitions, the rest-activity cycles or physical exercise [28], the fasting-eating cycles or nutrient intake as well as particular sleep stages [29] for instance. The master circadian clock has plasticity in function [30] in order to coordinate this information and to pass it downstream and outwards, for instance to sense metabolic cues [31] and to control the rest-activity cycles [32]. In individuals having seasonal variations in mood and behavior, the circadian clockwork tends to produce elastic rest-activity cycles [33], suggesting abnormalities in the reset of oscillations.

Seasons challenge the circadian pacemaker functions, because the information and feedback received change. A change of seasons may challenge a switch between the metabolic and circadian-based time-keeping mechanisms of action. Light-dark transitions are needed for the reset of the master circadian clock on a daily basis. When these signals are missed like it occurs with the decreasing photoperiod in general and the shortage of light exposure in the morning hours during winter in particular, the circadian clock tends to delay and the circadian clockwork may start relying more on the metabolic cycles producing time-giving signals needed for adaptation [34]. Fall and spring are the periods of the year which challenge the circadian clockwork and its integration. Studies in animals have demonstrated that in spring the morning-tagged or morning-active cells yield the dominance to the evening-active cells [35], or from the wake-up to sleep onset process, within the master circadian clock. This may need the input from the intrinsic clock which reacts to a change of seasons and drives its targets [36].

In individuals having seasonal variations in their mood and behavior, physical activities are usually reduced, whereby the effect of exercise on and the feedback from a peripheral circadian clock of the skeletal muscle [37] to the master circadian clock are altered. This may predispose to delays in the circadian clockwork. In addition, carbohydrate craving in the evening is a usual sign [38], which may lead to delays in the circadian clockwork [39]. In patients with seasonal affective disorder, there are also increases in the resting metabolic rate [40] during a depressive episode in winter. Concerning a plausible network or a potential pathway that might link the light-exposure and food-intake responsive oscillators to a range of affective and behavioral outputs, the mechanisms of action remain to be elucidated at molecular level.

We have demonstrated that the seasonal variations in mood and behavior in general and that those in weight and appetite in particular predispose to the metabolic syndrome. Our findings herein extend the seasonal pattern to the seasonal variations in mood and behavior that are part not only of depressive but also anxiety and alcohol use disorders. In addition, we characterize the metabolic component of their comorbid conditions in reference to the general population aged 30 and over living in Finland. Depressive and alcohol use disorders antithetical loadings on the metabolic and mental factors but an equal contribution to the degree of a problem experienced due to seasonal variations in mood and behavior.

A key question then is which factors have relevance to the occurrence of seasonal variations in mood and behavior. First, it has been reported that concerning those aged over 35 there is a positive correlation between latitude and the seasonal variations in mood and behavior [41]. We tested this association in our study herein. The latitude of residence or area of living had no significant effect on the intensity of seasonal variations or on the extent of a problem experienced due to the seasonal variations. Our current finding agrees with the data derived from European Union in contrast to United States [4], suggesting that migration and the way of life may influence the self-report. Second, the male sex and older age have been associated with a higher prevalence with a seasonal pattern among those with major depression [14]. We found the opposite herein so that there was a negative correlation between age and the seasonal variations in mood and behavior. Third, the sex acting through estrogen-related actions [42–44] or sleep durations [45] for example may play a role and give feedback on the master circadian clock. Furthermore, we found that taking physical exercise as well as drinking alcohol did affect the report, and our findings were consistent concerning their effects on seasonal variations in mood and behavior. Leisure time exercise habits as well as alcohol use can be considered as a secondary response, or alternatively as a primary choice of strategy, in order to cope with reoccurring changes in mood and behavior year after year. The former appears to buffer from distress experienced due to the seasonal variations, the latter tends to end at a problem. In trials with physical exercise, the positive effect on mood is easily compromised with the increasing intake of alcohol [46].

Our results herein give support to the hypothesized links between the metabolic, circadian, and seasonal cycles generated and guided by the master circadian clock. Moreover, our results of a general population now extend these links to include mood states as well. Our current results suggest that at population level benefits might be gained for the primary prevention of mood, anxiety, and alcohol use disorders. Interventions for the secondary prevention in the affected may exploit the combination of physical exercise with other modes of treatment [47], which seems to be effective. In order to have gains in favor of mental health, attention needs to be paid to the individual's leisure time exercise and drinking habits. A population-based approach at large, on one hand, to reducing alcohol misuse and, on the other hand, to augmenting physical exercise is appealing. This strategy needs to be realized not only on season's basis, but also in the long-term, and primary-care services are best placed to identify these problems and start specific interventions. Our problem-focused analysis herein identifies prevention challenges and provides their rank order.

A limitation is that we assessed the adequacy of lighting with the self-report of two items only, as part of the interview though. These two questions may not correlate linearly with the measured levels of lighting and luminance and therefore cannot be taken as any precise estimate of light exposure to the face and the eyes, but the two questions are meaningful in terms of disadvantage and hazard that are related to lighting conditions in the everyday life. Another limitation is that the seasonal variations in mood and behavior by using a self-report in our study. This questionnaire is retrospective to the routine seasonal variations during lifetime and has been evaluated to have a high internal consistency [48] and two-month test-retest reliability [49]. Our current analysis not only confirmed its good psychometric properties but also indicated that the global seasonality score was composed of two separate factors having relevance to mood and energy metabolism. On the other hand, this questionnaire has an advantage, as it focuses directly on the seasonal variations. It can be used as a sensitive screening instrument for general changes in mental health by season in the general population [50]. However, it is not reliable for comparison between populations of different geographic areas [51]. It is a valid psychometric screen to monitor seasonal symptoms in mental disorders, by providing traits of mood and behavior and by yielding a global score on how seasonal the disorders are.

Health-related seasonal phenomena are common and may have an impact of relevance on the health status of a population at large. Adaptation to the habitat seems to play a substantial role in northerly populations [52, 53], but there are also nonsurvivors [54]. Our results herein show now clearly that the latitude of residence had no effect on the degree of seasonal variations. They disagree with some previous survey reports but strengthen the view that the seasonally experienced problems in mood or behavior do not increase with increasing northerly latitudes. This means that other factors, for example signals or factors driving the transcription of circadian clock genes, may have a more powerful effect on the incidence than latitude or photoperiod. Understanding of the mechanisms of action underlying the seasonal variations in mood and behavior and their regulation needs and deserves further elucidation.

## Figures and Tables

**Table 1:** Determinants of the metabolic and mental factors of the Global Seasonality Score on Seasonal Pattern Assessment Questionnaire.

	Global Seasonality Score							
	The metabolic factor				The mental factor			
Explanatory variable	Estimate	Standard error	*t* value	*P* value	Estimate	Standard error	*t* value	*P* value
Waist circumference	0.0072	0.00095	7.59	<.0001	−0.0011	0.00093	−1.16	.25
Pelvic circumference	0.015	0.0016	9.46	<.0001	0.0019	0.0013	1.48	.14
Female sex	reference				reference			
Male sex	−0.20	0.025	−8.01	<.0001	−0.14	0.023	−6.29	<.0001
Normal self-reported lighting at home	reference				reference			
Poor self-reported lighting at home	0.41	0.097	4.20	<.0001	0.14	0.084	1.62	.11
Low GHQ-12 sum score	reference				reference			
High GHQ-12 sum score	0.17	0.036	4.65	<.0001	0.52	0.037	13.80	<.0001
Low BDI sum score	reference				reference			
Medium BDI sum score	0.13	0.030	4.21	<.0001	0.34	0.029	11.73	<.0001
High BDI sum score	0.31	0.056	5.48	<.0001	0.72	0.051	14.11	<.0001
No metabolic syndrome	reference				reference			
Metabolic syndrome	0.15	0.030	4.94	<.0001	0.020	0.024	0.85	.40
No major depressive disorder	reference				reference			
Major depressive disorder	0.24	0.065	3.70	.00022	0.49	0.047	10.43	<.0001
No diagnosis	reference				reference			
Alcohol use disorder	−0.00085	0.075	−0.011	.99	0.37	0.055	6.64	<.0001
Depressive disorder	0.21	0.071	2.90	.0037	0.47	0.054	8.73	<.0001
Anxiety disorder	0.058	0.085	0.68	.50	0.31	0.072	4.26	<.0001
Comorbid condition	0.25	0.11	2.35	.019	0.74	0.092	8.03	<.0001

GHQ-12 = 12-item General Health Questionnaire; BDI = Beck Depression Inventory.

**Table 2:** Determinants of the metabolic syndrome according the ATP-III criteria.

Explanatory variable	Estimate	Standard error	Odds ratio	95% confidence interval
Metabolic factor score	0.17	0.035	1.18	1.10 to 1.26
Aged 30 to 45	reference			
Aged 46 to 60	0.81	0.074	2.24	1.94 to 2.59
Aged 61 to 75	1.26	0.084	3.51	2.98 to 4.14
Aged 76 to 99	1.61	0.10	5.03	4.12 to 6.13
Low education level	reference			
Medium education level	−0.66	0.062	0.52	0.46 to 0.59
High education level	−1.00	0.074	0.37	0.32 to 0.43
Living alone	reference			
Living with someone	−0.24	0.057	0.79	0.70 to 0.88
Low exercise level	reference			
Medium exercise level	−0.48	0.054	0.62	0.56 to 0.69
Fitness training level	−0.84	0.088	0.43	0.36 to 0.51
Sports activities level	−1.27	0.29	0.28	0.16 to 0.49
Low alcohol use	reference			
Medium alcohol use	−0.42	0.070	0.66	0.58 to 0.76
High alcohol use	−0.57	0.090	0.57	0.47 to 0.68
No alcohol use	0.44	0.083	1.55	1.32 to 1.83
Low activity outdoors	reference			
Medium activity outdoors	−0.45	0.075	0.64	0.55 to 0.74
High activity outdoors	−0.61	0.073	0.54	0.47 to 0.62
Low BDI sum score	reference			
Medium BDI sum score	0.28	0.069	1.33	1.16 to 1.52
High BDI sum score	0.47	0.11	1.60	1.30 to 1.96

BDI = Beck Depression Inventory.

**Table 3:** Determinants of major depressive disorder according the DSM-IV criteria.

Explanatory variable	Estimate	Standard error	Odds ratio	95% confidence interval
Metabolic factor score	0.30	0.068	1.35	1.18 to 1.54
Mental factor score	0.65	0.063	1.91	1.69 to 2.16
Male sex	reference			
Female sex	0.68	0.15	1.98	1.49 to 2.64
Living alone	reference			
Living with someone	−0.53	0.12	0.59	0.46 to 0.75
Low social activity level	reference			
Medium social activity level	−0.56	0.15	0.57	0.43 to 0.76
High social activity level	−0.57	0.15	0.57	0.42 to 0.76
Low GHQ-12 sum score	reference			
High GHQ-12 sum score	2.21	0.13	9.16	7.16 to 11.71
Low BDI sum score	reference			
Medium BDI sum score	1.43	0.16	4.19	3.05 to 5.74
High BDI sum score	2.81	0.16	16.65	12.07 to 22.95

GHQ-12 = 12-item General Health Questionnaire; BDI = Beck Depression Inventory.

**Table 4:** Determinants of seasonal variations in mood and behavior in a representative sample of the Finns aged 30 and over.

Explanatory variable	Estimate	Standard error	*t* value	*P* value
Pelvic circumference	0.0050	0.00093	5.35	<.0001

Female sex	reference			
Male sex	−0.14	0.018	−7.86	<.0001
Low education level	reference			
Medium education level	0.13	0.021	6.19	<.0001
High education level	0.15	0.020	7.75	<.0001
Low GHQ-12 sum score	reference			
High GHQ-12 sum score	0.12	0.025	4.74	<.0001
Low BDI sum score	reference			
Medium BDI sum score	0.22	0.020	11.33	<.0001
High BDI sum score	0.34	0.037	9.23	<.0001
No major depressive disorder	reference			
Major depressive disorder	0.084	0.037	2.24	.026

GHQ-12 = 12-item General Health Questionnaire; BDI = Beck Depression Inventory.

**Table 5:** Degree of the metabolic factor based on seasonal variations in mood and behavior in a representative sample of the Finns aged 30 and over.

	Global Seasonality Score			Ratio of the metabolic to mental factor loadings			
	Mean	95% CI	S.D.	Mean	95% CI	S.D.	Median
No diagnosis	4.87	4.79 to 4.96	2.97	0.20	−0.14 to 0.55	12.20	0.25
Alcohol use disorder only	5.86	5.43 to 6.28	2.90	−0.17	−1.27 to 0.93	7.49	−0.32
Depressive disorder only	6.56	6.16 to 6.96	3.19	0.034	−2.44 to 2.50	19.82	0.39
Anxiety disorder only	5.95	5.34 to 6.56	3.33	0.41	−1.17 to 1.99	8.67	0.41
Comorbid condition	7.50	6.86 to 8.13	3.55	0.36	−0.47 to 1.19	4.63	0.41

CI = confidence interval; S.D. = standard deviation.

**Table 6:** Determinants of the seasonal variations experienced as a problem in a representative sample of the Finns aged 30 and over.

Explanatory variable	Estimate	Standard error	Odds ratio	95% confidence interval
Female sex	reference			
Male sex	−0.14	0.080	0.87	0.74 to 1.01
Living alone	reference			
Living with someone	−0.12	0.086	0.89	0.75 to 1.05
Normal self-reported lighting at home	reference			
Poor self-reported lighting at home	0.92	0.29	2.52	1.43 to 4.43
Low GHQ-12 sum score	reference			
High GHQ-12 sum score	0.53	0.11	1.71	1.37 to 2.13
Low BDI sum score	reference			
Medium BDI sum score	1.11	0.087	3.05	2.57 to 3.61
High BDI sum score	1.63	0.15	5.12	3.81 to 6.89
No diagnosis	reference			
Alcohol use disorder	0.67	0.18	1.96	1.37 to 2.80
Depressive disorder	0.78	0.15	2.19	1.64 to 2.93
Anxiety disorder	0.68	0.25	1.97	1.21 to 3.19
Comorbid condition	1.17	0.22	3.21	2.09 to 4.94

GHQ-12 = 12-item General Health Questionnaire; BDI = Beck Depression Inventory.
